# *Caenorhabditis elegans* glia modulate neuronal activity and behavior

**DOI:** 10.3389/fncel.2014.00067

**Published:** 2014-03-14

**Authors:** Randy F. Stout Jr., Alexei Verkhratsky, Vladimir Parpura

**Affiliations:** ^1^Department of Neuroscience, Albert Einstein College of MedicineBronx, NY, USA; ^2^Faculty of Life Sciences, The University of ManchesterManchester, UK; ^3^IKERBASQUE, Basque Foundation for ScienceBilbao, Spain; ^4^Department of Neurosciences, University of the Basque Country UPV/EHULeioa, Spain; ^5^Department of Neurobiology, Center for Glial Biology in Medicine, Civitan International Research Center, Atomic Force Microscopy and Nanotechnology Laboratories, and Evelyn F. McKnight Brain Institute, University of AlabamaBirmingham, AL, USA; ^6^Department of Biotechnology, University of RijekaRijeka, Croatia

**Keywords:** glia, evolution, behavior, invertebrate, *Caenorhabditis elegans*

## Abstract

Glial cells of *Caenorhabditis elegans* can modulate neuronal activity and behavior, which is the focus of this review. Initially, we provide an overview of neuroglial evolution, making a comparison between *C. elegans* glia and their genealogical counterparts. What follows is a brief discussion on *C. elegans* glia characteristics in terms of their exact numbers, germ layers origin, their necessity for proper development of sensory organs, and lack of their need for neuronal survival. The more specific roles that various glial cells have on neuron-based activity/behavior are succinctly presented. The cephalic sheath glia are important for development, maintenance and activity of central synapses, whereas the amphid glia seem to set the tone of sensory synapses; these glial cell types are ectoderm-derived. Mesoderm-derived Glial-Like cells in the nerve Ring (GLRs) appear to be a part of the circuit for production of motor movement of the worm anterior. Finally, we discuss tools and approaches utilized in studying *C. elegans* glia, which are assets available for this animal, making it an appealing model, not only in neurosciences, but in biology in general.

## Introduction: a brief on evolution of neuroglia

“Nothing in biology makes sense except in the light of evolution”.

Theodosius Dobzhansky (1900–1975)

Evolution of the nervous system proceeded through an increase in number and complexity of the nervous elements and through their specialization into electrically excitable neurons connected through defined synaptic contacts and electrically non-excitable neuroglia forming networks through intercellular gap junctions. Intercellular chemical neurotransmission is, however, characteristic for both forms of the neural cells that express appropriate receptors and are capable of secreting neurotransmitters. The evolution of the nervous system was not a straight journey from less complex and accomplished networks to the more refined ones; at the turning point between invertebrates and vertebrates, a fundamental metamorphosis occurred that changed the overall structure of the central nervous system (CNS). This change is associated with an appearance of radial glia, that in the vertebrates, serve as a universal neural precursor and a guide for neural cells to migrate through the thickness of the neural tube thus creating a layered organization of the CNS (Kriegstein and Alvarez-Buylla, 2009). This layered organization is at odds with the CNS of invertebrates that essentially appears as fused neural ganglia. This major metamorphosis in the CNS organization also coincided with an extinction of the whole class of cells highly elaborated in the invertebrates—the parenchymal neuroglia. Indeed, in hemichordates (that barely have any CNS) glial cells seem to be completely absent, whereas in the primitive vertebrates (such as, for example, zebra fish or certain sharks and rays) the CNS contains essentially one functional layer that could be completely penetrated by radial glial cells, which perform major homeostatic functions. Increase in the thickness and size of the CNS strained the radial glia, and a new wave of evolution of parenchymal neuroglia began in some Elasmobranchi with “elaborated” brain (Reichenbach et al., 1987; Ari and Kálmán, 2008). This evolution rapidly resulted in high diversification of neuroglia, which assumed all major homeostatic and many defensive responsibilities in the mammalian brain. However, the direction of neuroglial evolution in Chordata is rather similar to that in the invertebrates, in which neuroglia similarly underwent remarkable morphological and function diversification while climbing the phylogenetic ladder from the most primitive bilateralia to the arthropods with their well-developed nervous system (for detailed account on glial evolution and references see Hartline, 2011; Verkhratsky and Butt, 2013).

The early evolutionary history of neuroglia is complex and is far from being characterized in detail. There is a general agreement that supportive neural cells are absent in the diffuse nervous system of Cnidaria and Ctenophora, although there are unconfirmed reports about the existence of glia-like cells in the ganglia of scyphomedusae (Bullock and Horridge, 1965). Supportive neural (glia-like) cells are present in the nervous system of Acoelomorpha that are generally considered as the first bilateralia (Bery et al., 2010). Glial cells are found in Nematoda (Heiman and Shaham, 2007), but are absent in phylogenetically more advanced Bryozoa and Gnathifera/Rotifera, even though the Rorifera have a proper CNS, in which neuronal structures are surrounded by either epithelial or muscle cells (Wallace and Smith, 2013). Nonetheless, in Annelida and Arthropoda the neuroglia are well defined and diversified; glial cells become responsible for homeostasis of the nervous system, they provide the hemolymph-brain barrier, they are capable of mounting astrogliotic response to insult and they create ancestral myelin-like sheath around axons (Deitmer et al., 1999; Edwards and Meinertzhagen, 2010). Here, we focus on recent research on the neuroglia of the nematode,* Caenorhabditis elegans*, with particular attention to reports of glia modifying neuronal activity and behavior of this round worm, along with discussion of advantages and limitations of studying glia in the worm, particularly for neuron-glial interactions outside of development.

## Characteristics of *C. elegans* glia

We provide only a brief overview of the general properties of *C. elegans* glia, as the detailed information can be found in recent reviews in respect to roles in development (Shaham, 2005, 2006; Oikonomou and Shaham, 2011) and evolutionary aspects of worm glia (Heiman and Shaham, 2007).

### The exact number of glia in the worm

Early studies of the nervous system of* C. elegans* produced extremely detailed and meticulously categorized structural information allowing identification of each neural cell (Ward et al., 1975; White et al., 1986; Hall and Russell, 1991). This makes *C. elegans* one of the few animals whose full complement of individual cells has been mapped throughout development and the only such animal widely used as a model in neuroscience. The neuroglia of *C. elegans* were described based on light and electron microscopy, i.e., (ultra)structural characteristics of these cells; they appear to be a part of the nervous system, but did not have morphological characteristics of neurons, i.e., lack pre-synaptic structures (Ward et al., 1975; Thomas, 1994). At that time, a set of 56 cells were classified as glia-like support cells in hermaphrodites (Altun and Hall, 2010). Developmental lineage maps further supported this classification; 50 of the 56 glia-like cells were shown to be of the ectodermal lineage. Of note, the “Handbook of Worm Anatomy” section of the website www.wormatlas.org provides an overview of the anatomic and developmental characteristics of the glia of *C. elegans* in the “support cells” sub-section on the hermaphrodite nervous system.

### An ephemeral comparison of worm and mammalian glia

It is tempting to compare the glia of the worm to those of animals possessing more complex nervous systems. For these comparisons to be accurate, it is important to keep in mind that the nervous system of the worm likely contains fewer cells and connections that are optimal for its ecological niche, and not because it did not have enough evolutionary time or flexibility to attain greater complexity. Worms are not in possession of genes homologous to encoding glial fibrillary acidic protein (GFAP), a marker of astrocytes in mammals (although many other invertebrates express GFAP). Clearly, the functions that mammalian astrocytes perform in controlling blood flow and in contributing to the blood-brain barrier will not be possible for worm glia. Indeed, these functions are not required in the worms due to the small body size and characteristics of the environment in which they live. It is currently unknown if other functions of mammalian glia such as K^+^ clearance, and vesicular release and re-uptake of neurotransmitters are performed by glial cells of the roundworm.

Some genetic pathways for glial specification and development do seem to be shared between glia of mammals and those of *C. elegans.* The transcription factor LIN-26** was found to be required for glial cell development and ablation of the *lin-26* gene may cause cells that would become sheath glia to take on some characteristics of neurons (Labouesse et al., 1994). This was the first in a series of genetic/developmental findings that should be considered when we think about how glia evolved in different species. For example, the *hlh-17* gene promoter has been used as a marker for the cephalic (CEP) sensilla sheath (CEPsh) glia (McMiller and Johnson, 2005). The *hlh-17* gene seems to be important for development, but not initial specification of the CEPsh glia (Yoshimura et al., 2008). The *hlh-17* gene has homology to the mammalian regulator of glial development *Olig2*. Genes that regulate the dorsal/ventral patterning of OLIGodendrocyte lineage transrctiption factor 2 (OLIG2) expression in mammals share homology to those required for normal HLH-17 expression specifically in the dorsal CEPsh glia of the worm (Yoshimura et al., 2008 and as reviewed in Oikonomou and Shaham, 2011).

### Worm glia are unnecessary for neuronal survival

Based on experiments using gene deletions and/or cell ablations of glial precursors during development, we do know that a major difference between nervous system of *C. elegans* and nervous system of more advanced animals is that glia are mostly not required for survival of neurons within the mature nervous system of the worm and also the worms are able to survive and reproduce without glia. Except for the notable exceptions discussed later in this review, the glia of the worm seem to function mainly in guiding development of sensory structures in the worm and then act as a barrier by ensheathing the sensory structures in the adult worm. We know a great deal about how genes modulate the development and activity of *C. elegans* neurons, and also the neural molecular components that are shared (and not shared) between the neurons of *C. elegans, Drosophila*, mice, and human. Examining the role of *C. elegans* glia in neural function may help us to understand of how these cells modulate neuronal activity or behavior in order to compare roles that glia of *C. elegans* and mammals play in information processing (see below).

### Worm glia play a role in proper development of sensory organs

Early ablation studies indicated that the sheath and socket glia played a role in the development of the ciliated sensory ending (Bargmann et al., 1990; Vowels and Thomas, 1994). The tractable genetics and other advantages of the worm were used to show that the glia of *C. elegans* affected sensory activity by controlling the development of cellular compartments surrounding sensory cilia (reviewed in Procko and Shaham, 2010). All of the ectoderm-derived glial cells of the worm are associated with the endings of these sensory neurons; neuronal dendritic endings together with glia form sensory organs of the worm known as sensilla. These specialized structures are prominent aspects of the nematode nervous system, and they fail to develop correctly without normal neuron-glia interactions. Neuronal development and maintenance of sensory structures require not only a set of genes expressed in the neurons, but also glial specific genes (reviewed in Oikonomou and Shaham, 2012). Attachments for sensory dendrites during migration of neurons during development also require factors released by the glia of *C. elegans* (Heiman and Shaham, 2009). Furthermore, it was recently suggested that the sensory synapse of the worm could be used as a model to study neuron-glia interactions in the human CNS (Shaham, 2010), albeit the usefulness of this model in this context remains to be seen.

Neuroglia in *C. elegans* perform at least four broad roles in the nervous system: (1) establishment of the location of neuronal structures; (2) regulation of sensory ending size and morphology; (3) a barrier that bundles and separates neuronal elements from other cells; and (4) modulation of neuronal activity. In a certain way this quartet resembles general roles that vertebrate glia are thought to have in the central and peripheral nervous systems. The latter two roles, interrelated with some worm cells and behaviors, are further discussed below.

## The CEP sheath glia in synapse maintenance and dopamine-linked behavior

**Figure 1:**
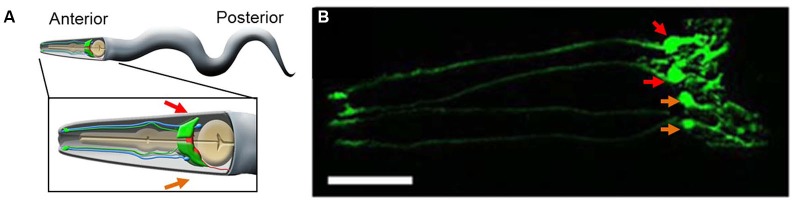
**The CEPsh glia. (A)** A cartoon of an adult worm showing the four CEPsh glial cells (green) positioned in the anterior of the worm (inset). The CEPsh cell bodies with their velate extensions are positioned around the central nerve ring (red) which they enwrap along with the proximal section of the ventral nerve cord. Additionally, each CEPsh glial cell possesses a long anterior process, emanating to the anterior sensory tip, which closely interacts with the dendritic extension of a nearby CEP neuron (blue). Arrows indicate the dorsal (red arrows) and ventral (orange arrows) side of the worm. **(B)** A confocal image showing green fluorescent protein (GFP) expression driven by the *hlh-17* promoter to visualize the four CEPsh glial cells (worm strain VPR839). The anterior (head) of a juvenile (larval stage 4) worm is shown; the worm is turned ∼45^˚^ from “upright” such that all four CEP sheath cells are visible. The sheath portion of the cells that form a tube around the dendritic endings of the CEP neurons are seen at the left of the image. The dorsal (red arrows) and ventral (orange arrows) CEPsh cell bodies are seen. The thin sheet-like extensions that surround and invade the nerve ring are seen in the rightmost part of the image. Scale bar, 20 μm. Image adapted from Stout et al. (2013).

As already implicated, there appears to be a special class of cells among worm glia. We start our discussion by describing the four CEPsh glial cells (Figure 1). These ensheathing cells form a tubular structure surrounding the anterior tip of the sensory ending of CEP neurons and are therefore categorized into the group of 24 sheath glia found in the anterior of the worm. The CEPsh cells are unique in this glial pack in that they also extend thin sheet-like processes which ensheath the nerve ring, i.e., the worm “brain”. Thin CEPsh cell processes also extend into the neuropil (White et al., 1986; Durbin, 1987; Oikonomou and Shaham, 2011).

There is morpho-functional heterogeneity between worm glia as in other animals. The morphology of the four CEPsh glia differs substantially from that of other sheath and socket glia (see below). The two ventral CEPsh cells express netrin (uncoordinated-6) but expression of this neuroligand involved in axon guidance is not detected or required in the dorsal pair of CEPsh glia (Hedgecock et al., 1990; Wadsworth et al., 1996; Yoshimura et al., 2008). Thin velate protrusions of the membrane of the CEPsh cells into the nerve ring appear to have some specificity as they are seen in proximity of the same neuronal synapses across different individual worms examined by electron microscopy (Ward et al., 1975; Durbin, 1987). Indeed, the CEPsh cells regulate synapse location through expression of the worm homolog of mammalian netrin (Colón-Ramos et al., 2007). More generally, the CEPsh glia are required for the maintenance of synaptic connectivity within the nerve ring (Shao et al., 2013, and reviewed in Yates, 2013).

Exciting, although circumstantial, evidence indicates that the CEPsh cells modulate dopamine-dependent behaviors in the worm, including feeding and a form of learning (Felton and Johnson, 2011). The *hlh-17* gene encodes the basic Helix-Loop-Helix transcription factor HLH-17 that is expressed almost exclusively in the CEPsh cells (McMiller and Johnson, 2005; Yoshimura et al., 2008). Disruption of the *hlh-17* gene led to changes in egg-laying behavior, feeding behavior-plasticity deficits, and impaired a form of gustatory associative learning. The four CEPsh glia are closely associated with the four CEP neurons, which help mediate the aforementioned behaviors through release and up-take of dopamine (other neurons and neurotransmitters are mediators as well). Although the *hlh-17* gene is not required for development/survival of the CEP neurons and sheath cells, the gene is required for dopamine-dependent behaviors as the loss of *hlh-17* somehow affects dopamine signaling between the CEP neurons. These data represent an exciting hint that CEPsh glia modulate dopamine signaling and future research into this area of worm neurobiology is highly anticipated, especially in light of the role for dopaminergic signaling and dopamine transporters in human neurological diseases.

## Amphid sheath and socket glia tune sensory neuron activity and sensory behavior

**Figure 2:**
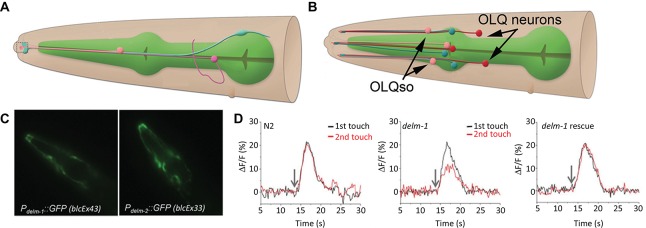
**The amphid sheath and outer labial sensilla socket cells. (A)** The amphid sheath glia (blue) cell body is positioned near the nerve ring (not shown) and sends a long, thin process, along with a neuronal dendrite (magenta) and the amphid socket cell process (pink) to the anterior tip of the worm (left side, dashed box); adapted from *wormatlas.org*. **(B)** A cartoon showing the outer labial sensilla sheath (OLLsh and OLQsh, blue) and socket (OLLso and OLQso, pink) cells and their extensions to the anterior of the worm where they ensheath the ciliated dendrite of the neurons (OLL and OLQ, red); note that inner labial sensilla is not shown here; adapted from *wormatlas.org*. **(C)** The promoters for the *delm-1* and *delm-2* drive reporter GFP expression in the outer and inner labial sensilla socket glial cells (OLQso and ILso, respectively) of worms (strains blcEx43 and blcEx33, respectively). **(D)** Knock out of *delm-1* leads to reduced OLQ neuron calcium response to mechanical stimulation of the worm (middle), while re-expression of the channel in OLQso glia (using the glial promoter *itx-1*) rescued the neuronal responsiveness (right); N2, background strain (left). **C** and **D** adapted from Han et al. (2013).

There is strong evidence that channel activity within other (than CEPsh) sensilla-associated glia modulates neuronal activity, which in turn, affects behavioral responses to environmental stimuli (Wang et al., 2008, 2012; Han et al., 2013). These glia form sheaths around bundles of ciliated sensory dendrites (known collectively as the amphid inner and outer labial sensory organs) at the anterior tip of the worm. Promoter-reporter approaches mapped expression of several DEGenerin/Epithelial Na^+^ Channels (DEG/ENaC) class channels to the sheath and socket glia. Through glial specific re-expression of DEG/ENaC channels ACid-sensitive Degenerin (ACD)-1 and DEgenerin Linked to Mechanosensation (DELM)-1,2 it was shown that expression of these channels in sheath and/or socket glia modulates the activity of sensory neurons (Wang et al., 2008, 2012; Han et al., 2013). The ACD-1 channel is required in the amphid sheath glia (depicted in Figure 2A), while the DELM-1 and 2 channels act in the inner and outer labial socket glia (depicted in Figure 2B). The DELM-1 and -2 are required in the glia but not in neurons in order for the worm to perform a set of foraging related behaviors. In the cells expressing the DELM-1 and 2 channels only re-expression under a glial socket cell promoter rescued sensory behavior and neuronal calcium signaling deficits (Figures 2C, D). Similarly the effects of *acd-1* knockout were only rescued by re-expressing the gene in the amphid sheath glia.

So, how could expression of an ion channel in glia modulate sensory neuron activity and mechanosensory behavioral responses? It has been postulated that activity of glial DEG/ ENaC channels leads to an increase in extracellular K^+^ and thereby to an increase in excitability of the nearby neuronal processes. There is some semblance of this mechanism to specialized regulation of K^+^ concentration at sensory endings of vertebrates (Pacinian corpuscle), which modulates sensory neuron activity (Hyinsky et al., 1976). In another study, calcium responses of chemosensory neurons (Amphid Wing Cell (AWC) neurons) to chemical (isoamyl alcohol) stimulations near the detection threshold were used to show that loss of the glial specific ACD-1 channel is required for normal AWC neuron calcium response, but that ACD-1 is not directly affected by such stimulation. Artificial manipulation of the baseline activity of the AWC neurons by expressing a human capsaicin-sensitive TRPV1 channel in the worm’s AWC neurons masked the effect of the loss of glial ACD-1. Finally, it was demonstrated that the ACD-1 channels were localized to the anterior end of the worm where the AWC sensory dendrites interacted with chemical stimulant. While all aspects of this neuron-glia interaction are not clear, available evidence supports the idea that the glia modulate the threshold for neuronal excitability. Furthermore, it seems that two different sets of glial cells modulate the activity of sensory synapses using different ion channels and that this action is specific to a subset of synapses within the same glial “cradle” structure, this latter being a concept put forward to characterize the role of astrocytes at the vertebrate synapse (Nedergaard and Verkhratsky, 2012). In conclusion, the CEPsh glia are important for development, maintenance and activity of central synapses, whereas the amphid glia seem to set the tone of sensory synapses.

## The GLRS: an unorthodox glial TYPE

**Figure 3:**
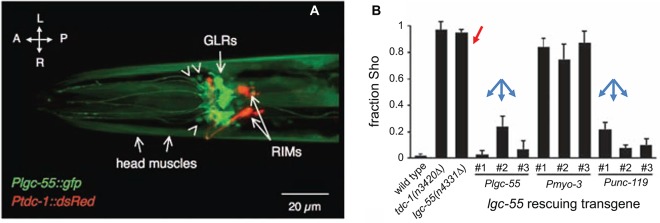
**LGC-55 expressed in the Glial-Like cells in the nerve Ring (GLRs) is required for normal tyraminergic modulation of head movements by *C. elegans*. (A)** The promoter for the tyramine receptor LGC-55 drives expression of GFP in the six GLRs and head muscle cells of *C. elegans*; RIMs, tyraminergic interneurons expressing red fluorescent protein. Orientation arrows, L-R, left-right, A-P, anterior-posterior. Arrowheads indicate some of the unidentified head neurons that express the *gfp* transgene. **(B)** Worms carrying a mutation in the *lgc-55* gene (or in the *tdc-1* gene, encoding tyrosine decarboxylase, an enzyme that converts L-tyrosine to tyramine) do not suppress foraging movements when crawling backward (Sho phenotype; red arrow). Re-expression of the LGC-55** driven by either the *lgc-55* or *unc-119* (pan-neural) promoters (blue arrows), but not by *myo-3* (muscle) promoter, rescues the behavioral phenotype, indicating that glia or neurons are responsible for the behavior. Adapted from Ringstad et al. (2009).

We next discuss the evidence for a signaling pathway between neurons to muscle cells that may pass through an unusual type of *C. elegans* glial cells. Namely, the six Glial-Like cells in the nerve Ring (GLRs) were named based on their morphology and location; however, unlike other worm glia the GLRs are of mesodermal origin (reviewed in www.wormatlas.org (Altun and Hall, 2009)). It is somewhat surprising that a cell type of different origin can so closely morphologically resemble all other glial cells of the worm. Because of the lineage, a comparison to mammalian microglia is tempting, especially in light of a GLR cell having been observed (by electron microscopy) apparently engulfing a dying CEP neuron (Nass et al., 2002). The GLRs are integrated into the nervous system of the worm and contribute to the development of the nerve ring and pharynx (muscle-based feeding organ of the worm). GLRs are connected to both neurons and muscle cells in the head by gap junctions and may be part of the circuit for producing specialized fine motor movements of anterior of the worm during foraging that are analogous to neck movements of vertebrates (White et al., 1986; Ringstad et al., 2009). Two reports examining the role of Ligand-Gated ion Channel (LGC)-55, a tyramine receptor, pointed to its expression in the GLRs. One report indicates that LGC-55 could function in GLRs or neurons to suppress foraging activity during backward movement (Ringstad et al., 2009; Figure 3). Another report points to the function of the LGC-55 in muscle and to a lesser extent in neurons (Pirri et al., 2009).

Potential involvement of GLRs in a circuit relevant for behavior is intriguing and warrants further research. This research, into the function of GLRs, however, has been hampered by the lack of a cell specific promoter. This challenge could potentially be circumvented with combinatorial-conditional promoter systems to limit genetically based markers, activity indicators, or functional modulators to the GLRs (Voutev and Hubbard, 2008). The finding that there are gap junctions between the GLRs and neurons is intriguing especially in light of recent discoveries that gap junctions seem to connect the neurons to glia of the sensory ganglia (Suadicani et al., 2010) and as reviewed by Hanani (2012) and Huang et al. (2013) and in the developing CNS (Pakhotin and Verkhratsky, 2005) of mice. The protein subunits that make up the gap junction channels of invertebrates (innexins) and vertebrates (connexins) share no protein sequence homology, but form similar macromolecular structures that connect the cytoplasm of adjacent cells, as reviewed in Scemes et al. (2007) and Simonsen et al. (2014). Additionally, the GLRs of the worm have taken on morphological characteristics of glia although they follow different developmental path, and express a different set of genes than other *C. elegans* glia. This may represent a case of compound convergent evolution and points to a role of gap junction based neuron-glia interaction as a rare but fundamental process, since it seems to have arisen independently in highly divergent nervous systems. Therefore, this may be a particularly important area for future study. The genome of *C. elegans* contains 25 genes for innexins (Starich et al., 2001); a promoter-reporter based survey indicated that the sheath and socket glia also express several innexin genes (Altun et al., 2009). This is another common feature shared between the CEPsh glia and vertebrate astrocytes—prominent expression of gap junctions. However, gap junctions connecting CEPsh glia to other cells have not been investigated and this is currently a woefully understudied aspect of worm glia.

The recently discovered/renamed cell type known as telocytes may be the mammalian equivalent of the worm GLRs (Gherghiceanu and Popescu, 2011; Cretoiu et al., 2012; Smythies and Edelstein, 2014). If the set of genes expressed by the GLRs turns out to share similarity to the set expressed in telocytes, perhaps the GLR-type should be reclassified from glia to ancestral telocytes. Although this is currently purely speculative, such classification would establish the GLRs as a model to study the biology of this exciting new vertebrate cell type.

Finally, it is worth mentioning that the GLRs are connected by gap junctions to ring motor neurons which themselves receive synaptic input from cells that make up a gap junction-mediated circuit with coincidence detection features (Rabinowitch et al., 2013). It will be important to test if the activity of the GLRs influence this or associated circuits and if gap junctions are involved in GLR physiology.

## Approaches in studying glia in *C. elegans*

**Figure 4:**
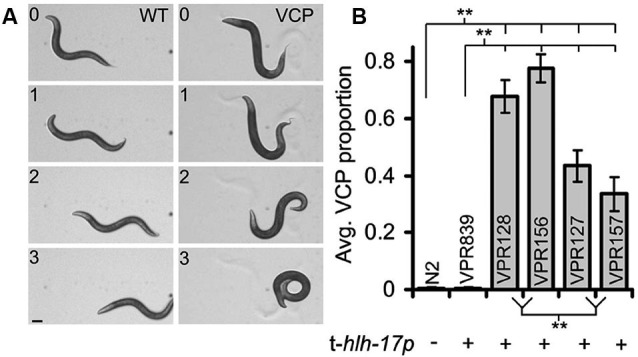
**A subset of *C. elegans* strains carrying transgenic arrays containing the trans-*hlh-17* promoter (t-*hlh-17p*) to drive expression of reporters in the CEPsh glial cells display a ventral coiler phenotype (VCP) during backward movement. (A)** A time-lapse montage of worms showing a normal/sinusoidal pattern during backwards locomotion (WT, left column). Worms with many copies of the t-*hlh-17p* coil when they attempt to crawl in reverse (VCP, right column; strain VPR156). Numbers indicate time in seconds. Scale bar, 100 μm. **(B)** Non-transgenic worms (WT, N2) do not display the VCP, but different strains of worms (VPR) containing the t-*hlh-17p*, except the VPR839 strain, display varying degrees of abnormal locomotion. ** Indicates a significant difference. Adapted from Stout et al. (2013).

Several themes emerge in the approaches used in the research highlighted above. Rescue of genetic ablation-induced changes to neuronal function or behavior through use of glia-specific promoters is widely used in both invertebrates and vertebrates. This approach is particularly powerful for *C. elegans* since an enormous number of mutant strains are available. The determined cell lineage and ease of specific cell identification is an advantage. Additionally, *C. elegans* is probably the easiest and cheapest animal model organism in which to produce and maintain transgenic animal lines. Cell-type specific promoters are available and, in combination with the phenomenon of transgene mosaicism, genetic rescue or other manipulations can be targeted to individual glial cells (Colón-Ramos et al., 2007; Yoshimura et al., 2008). Transgenes can affect neuronal and behavioral phenotypes in unexpected ways, but if such effects are detected they can be controlled for by using alternative strategies for transgene introduction (Kage-Nakadai et al., 2012; Stout et al., 2013). For example, worms carrying many (more than ~200) copies of the transgenic *hlh-17* promoter display a ventral coiling behavior during backward locomotion (Figure 4); coiling occurrences correlated to the transgene promoter copy number (Stout et al., 2013). This behavior is variable across different transgenic worm strains and can range from subtle to near complete inability for worms to crawl in the backward direction. If worm strains used in studies on worm behavior exhibit even a very mild form of the ventral coiling behavior it would be expected to have a major impact on sensitive behavioral tests such as salt-food associative learning and measures of reversal frequency or on measures of shape of body-bending during locomotion. Future studies where behavioral alterations are attributed to glia will need to control for the possibility that promoters used to target glial cells may cause (in)direct behavioral effects (Stout et al., 2013).

**Figure 5:**
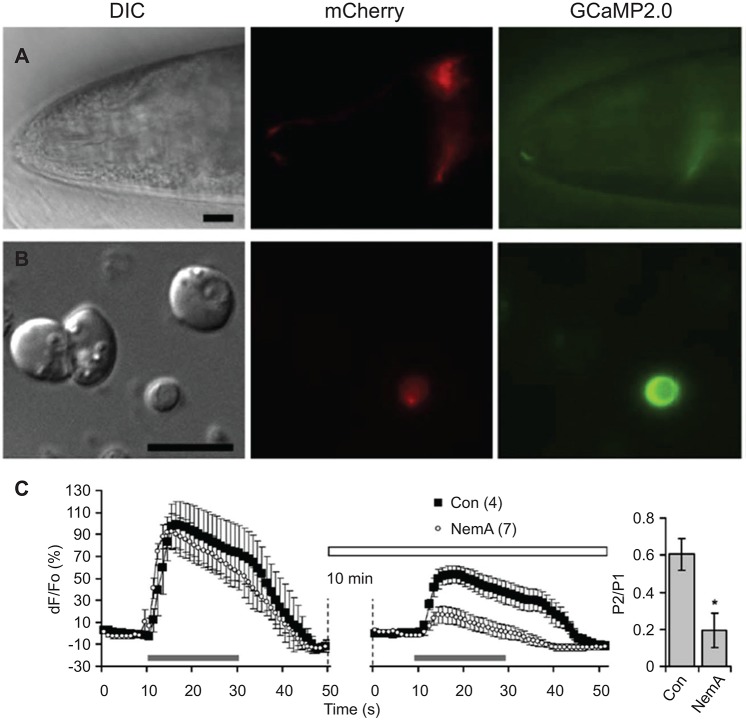
**L-type voltage-gated Ca^2+^ channels (VGCCs) play a role in depolarization-induced intracellular Ca^2+^ elevations in CEPsh glial cells. (A)** The *hlh-17* promoter can be used to drive expression of a red fluorescent protein marker (red, mCherry) in the CEPsh glia along with a fluorescent protein-based Ca^2+^ sensor (green, GCaMP2,0). Differential interference contrast (DIC). An anterior portion of an L4 stage worm (VPR108 strain) is shown. **(B)** CEPsh glial cells in mixed culture prepared from embryos can be identified based on their mCherry/GCaMP2.0 expression. **(C)** Time-lapse of GCaMP2.0 fluorescence emission from CEPsh glial cells. Paired-pulse application of a depolarization stimulus, high extracellular potassium (HiK^+^, 100 mM; horizontal grey bar) to CEPsh glial cells results in an elevation of intracellular Ca^2+^ levels (black squares). Nemadipine-A (NemA), a pharmacological L-type VGCC blocker, can be used to test the channels present in glial cells in culture (horizontal open bar); Con, sham stimulated control. (right, bar graph). Ratio of the peak Ca^2+^ level in response to the second HiK^+^ application (P2) over the first application (P1). * Indicates a significant difference. Adapted from Stout and Parpura (2011).

Optical methods for recording neuronal activity are currently more widely used than electrophysiological methods due to difficulty in accessing neurons with patch electrodes which is hampered by the worm cuticle and internal pressure (Kerr et al., 2000; Kerr and Schafer, 2006). Thus far, glial cell activity has only been assessed by genetically encoded optical indicators (Stout and Parpura, 2011; Wang et al., 2012). The ability to culture embryonic and adult stage *C. elegans* cells (Christensen et al., 2002; Frøkjaer-Jensen et al., 2006; Strange et al., 2007), including glia (Stout and Parpura, 2012; Sangaletti and Bianchi, 2013), should ease electrophysiological access to glial cells and has allowed acute application of pharmacological manipulations (Stout and Parpura, 2011; Figure 5). For instance, the combination of genetically encoded indicators, mutant strains carrying deletions of voltage-gated Ca^2+^ channels (VGCCs), and pharmacological treatments showed that cultured CEPsh glia respond to membrane depolarization with increases in intracellular Ca^2+^ mediated by various types of VGCCs (e.g., the role of L-type is shown in Figure 5). Optogenetic manipulations, i.e., use of channelrhodopsin, have been very useful in the study of *C. elegans* neurons (Nagel et al., 2005), but behavioral effects of light-activated channels have not been hitherto reported for *C. elegans* glia.

It may seem surprising that no reports of laser or genetic ablation of *all* CEPsh glia in *adult* worms have been published, particularly since post-embryonic, larval-stage ablation of a subset of the CEPsh cells led to interesting phenotypes. Briefly, when the precursor cells of the CEPsh glia are ablated during embryonic development the CEP neuron dendrites are shortened, axons in the nerve ring are disrupted, and the entire nerve ring is disrupted in some animals that lack the CEPsh glia. Some worms even failed to develop past the larval L1 stage when CEPsh glia were ablated (Yoshimura et al., 2008). Heat shock-inducible expression of a cell-killing caspase (Chelur and Chalfie, 2007) in adult CEPsh cells led to defects in synapse maintenance in the Amphid Interneuron Y (AIY), but incomplete ablation of all CEPsh glia across individual worms may have occurred (Shao et al., 2013). These data highlight the need for future research to further assess the effect of acute loss of CEPsh glia in adulthood. The present lack of experiments ablating the all CEPsh glia in adult worms, however, may be due to the unusual nature of the CEPsh glia in that they have a large cellular surface area that is spread over a large portion of the anterior nervous system.

In general, many of the methods that worked so well for the study of worm neurons have been harder to implement for glial biology. The development of new optogenetic probes and channels along with our rapidly increasing knowledge of gene expression in worm glia (Spencer et al., 2011) can be expected to make the discoveries we described in this review a start to an exciting time in research on glia-neuron interactions in the adult worm.

## Envoi

The intent of this focus review was to summarize current evidence indicating that the glia of *C. elegans* have an important role in modulation of neuronal activity and behavior. Indeed, future studies are needed to understand details of glial roles in the nervous system of this nematode. Meanwhile, *C. elegans* has proven enormously helpful in unveiling mysteries surrounding the operation of not only the nervous system, but also of many other basic biological functions. Consequently, it shall not come as a surprise that the National Institutes of Health list *C. elegans* as one of the model organisms for biomedical research.^1^ This “feather in the cap” has been earned as a consequence of *C. elegans* being one of the animals whose full complement of individual cells has been mapped throughout development and due to the ease of genetic manipulations, with rapid outcomes, which this nematode offers. Moreover, there are publicly available information resources (e.g., WormAtlas, WormBase and WormBook; http://www.wormatlas.org, http://www.wormbase.org, and http://www.wormbook.org, respectively), and consortia providing various mutation/knockout/transgenic strains on a thrifty budget (e.g., Caenorhabditis Genetics Center at University of Minnesota, Minneapolis, MN; http://www.cbs.umn.edu/research/resources/cgc), making *C. elegans* an appealing model in neurosciences and biomedicine.

## Conflict of interest statement

The authors declare that the research was conducted in the absence of any commercial or financial relationships that could be construed as a potential conflict of interest.

## Footnotes

^1^http://www.nih.gov/science/models/
